# Development and Application of a Triplex RT-qPCR Assay for Differentiating Major Lineages of Porcine Reproductive and Respiratory Syndrome Virus

**DOI:** 10.3390/ani16172642

**Published:** 2026-08-23

**Authors:** Tao Liu, Xiuwen Zhang, Qingan Han, Yuntao Liu, Yi Wang, Liang Hao, Yao Li, Peng Liu, Jinghui Fan

**Affiliations:** 1 College of Veterinary Medicine, Hebei Agricultural University, Baoding 071000, China; liutao93307@163.com; 2 Yanyun Laboratory, Baoding 071000, China; zhangxiuwen@casmed.cn (X.Z.); wangyi@casmed.cn (Y.W.); 3 College of Life Sciences, Hebei Agricultural University, Baoding 071001, China; 4 Hebei Key Laboratory of Analysis and Control of Zoonotic Pathogenic Microorganism, Baoding 071000, China; hanqa1970@126.com (Q.H.); 18233153723@163.com (L.H.); liyao@casmed.cn (Y.L.); 5 Hebei Provincial Center for Animal Disease Prevention and Control, Shijiazhuang 050035, China; 6 Ringpu (Baoding) Biopharmaceuticals Co., Ltd., Baoding 071000, China; yuntaoliu01@163.com; 7 CasVet Inc., Baoding 071000, China

**Keywords:** porcine reproductive and respiratory syndrome virus, swine, triple TaqMan-qPCR

## Abstract

Porcine reproductive and respiratory syndrome (PRRS) represents a critical infectious disease caused by the PRRS virus (PRRSV), posing a substantial threat to the global swine industry. In China, there is currently an epidemic trend characterized by the coexistence of multiple evolving genotypes. Effective prevention and control measures are contingent upon the availability of rapid, precise, and sensitive pathogen detection technologies. Addressing the need for swift differentiation of the predominant circulating strains, including the classical strains (PRRSV-C), the highly pathogenic strains (PRRSV-HP), and NADC30-like strains (PRRSV-NA), this study focuses on the NSP2 region of each lineage. It establishes a triple TaqMan-qPCR method capable of simultaneously genotyping these three lineages. The method demonstrated no cross-reactivity with other viruses, including porcine parvovirus (PPV), porcine transmissible gastroenteritis virus (TGEV), porcine pseudorabies virus (PRV), classical swine fever virus (CFSV), African swine fever virus (ASFV), porcine epidemic diarrhea virus (PEDV), porcine rotavirus (RV), and porcine circovirus (PCV2), thereby fully affirming its specificity. The sensitivity analysis demonstrated that the limit of detection (LOD) for the NSP2 gene in each lineage was 1 copy/μL based on the purified plasmids. Both inter-group and intra-group coefficients of variation (CV) were less than 4%, indicating high reproducibility. Comparative studies with commercial kits revealed that the developed TaqMan-qPCR method exhibited 100% relative sensitivity and a relative conformity rate exceeding 98%, suggesting its potential as a viable alternative to commercial kits. Furthermore, the analysis of 1049 clinical samples using the qPCR method indicated that the PRRSV-NADC30-like strains are currently the predominant circulating strain in clinical settings in Hebei Province. In conclusion, this study developed a triple TaqMan-qPCR method capable of simultaneously identifying PRRSV-C, PRRSV-HP and PRRSV-NA, enabling rapid and accurate identification of the PRRSV genotypes prevalent in pig populations. This provides a robust technical tool for the development of targeted immunization and prevention strategies.

## Simple Summary

Porcine reproductive and respiratory syndrome virus (PRRSV) is a major pathogen in the swine industry, presenting a longstanding and significant threat to global pig production. In China, there is currently an epidemic trend characterized by the coexistence of multiple evolving genotypes. To address the need for rapid differentiation of the predominant circulating strains, including the classical strains (PRRSV-C), the highly pathogenic strains (PRRSV-HP), and NADC30-like strains (PRRSV-NA), this study focused on the NSP2 region of each strain and established a triple TaqMan-qPCR method capable of simultaneously genotyping the three strains. This detection method has high specificity and shows no cross-reaction with various pig-susceptible viruses. Moreover, its relative detection accuracy compared to the mainstream commercial kits in the market exceeds 98%. By using the qPCR method to analyze 1049 clinical samples, it was found that the NADC30-like strains are currently the main prevalent strain of PRRSV in the clinical environment of Hebei Province. The triple TaqMan-qPCR method developed in this study can quickly and accurately identify the prevalent PRRSV genotypes in pig populations. This provides a reliable technical tool for formulating targeted immunization and prevention strategies.

## 1. Introduction

Porcine reproductive and respiratory syndrome virus (PRRSV) is a major pathogen in the swine industry, presenting a longstanding and significant threat to global pig production [1]. This virus is responsible for reproductive disorders and respiratory diseases, and it compromises the immune system of pigs, thereby increasing their susceptibility to secondary infections [2]. Consequently, PRRSV severely impacts the health and productivity of pig populations. According to the most recent taxonomic classification by the International Committee on Taxonomy of Viruses (ICTV), PRRSV is identified as a single-stranded positive-sense RNA virus within the order Nidovirales, family *Arteriviridae*, subfamily *Variarterivirinae*, and genus *Betaarterivirus*. The viral genome is approximately 15 kilobases in length and comprises at least 10 open reading frames (ORFs) [3]. Within the genus *Betaarterivirus*, two distinct viral species are officially recognized: *Betaarterivirus suid 1* (referred to as PRRSV-1, previously known as the European-type PRRSV, with the Lelystad virus serving as the prototype strain) and *Betaarterivirus suid 2* (referred to as PRRSV-2, previously known as the North American-type PRRSV, with VR-2332 as the prototype strain) [4]. These two species exhibit approximately 50–70% nucleotide sequence identity and display significant antigenic differences [5]. In China, PRRSV-2 constitutes the predominant circulating viral population, whereas PRRSV-1 infections have been reported only sporadically in a limited number of regions [6].

PRRSV exhibits a notably high mutation rate and recombination frequency, contributing to its extensive genetic diversity [7,8]. Utilizing the widely accepted phylogenetic classification system based on the ORF5 gene, global PRRSV-2 strains are classified into 11 distinct genetic lineages (Lineages 1–11) [9]. These lineages demonstrate nucleotide divergence ranging from 9.06% to 17.18%, with each lineage displaying unique geographical distribution patterns [9]. In China, the evolutionary trajectory of PRRSV-2 lineages has been thoroughly documented. During the period from the 1990s until approximately 2006, Lineage 5, exemplified by the prototype strains CH-1a and VR-2332 (commonly known as classical PRRSV), was the predominant lineage in circulation [10]. However, following the nationwide outbreak of highly pathogenic PRRSV (HP-PRRSV) in 2006, Lineage 8, represented by strains such as JXA1 and HuN4, swiftly became the dominant lineage throughout China [10]. Since its initial detection in China in 2013, Lineage 1, with NADC30 serving as the reference strain (also referred to as NADC30-like strains), has exhibited a steady increase in prevalence. This rise is attributed to its significant recombination capacity and adaptability in transmission. Consequently, Lineage 1 has supplanted Lineage 8 as the dominant circulating lineage within Chinese swine herds [11] and has spread extensively across several provinces [12,13]. Research has demonstrated that recombination events between the NADC30-like lineage and other PRRSV lineages are prevalent, contributing to the genetic diversity of the virus and resulting in pathogenic variations among different strains [14,15]. Consequently, China is currently experiencing the coexistence of classical strains, highly pathogenic variants, and NADC30-like strains, with frequent co-occurrence and recombination events across different lineages. This complexity exacerbates the challenges associated with virus transmission and pathogenicity [16,17]. The intricate epidemiological landscape of these variants not only complicates the molecular diagnosis of the virus but also imposes more stringent demands on vaccine development and control strategies.

Currently, the molecular detection of PRRSV predominantly utilizes reverse transcription quantitative polymerase chain reaction (RT-qPCR) methodologies, which are extensively employed in clinical diagnostics and epidemiological surveillance. Despite the development and application of various single- or dual-target detection techniques for the identification of distinct lineages, these methods frequently focus on a singular virus lineage or a narrow spectrum of variants. This limitation hinders the simultaneous and efficient detection of diverse PRRSV populations [18,19] and further constrains the timeliness and accuracy of early diagnosis and epidemic management. To address these limitations, it is essential to develop a multiplex molecular detection technology capable of simultaneously achieving high-sensitivity and high-specificity detection of PRRSV-C, PRRSV-HP, and PRRSV-NA. This study employs qPCR technology, utilizing specific primers and probe combinations targeting three distinct PRRSV variants. It systematically evaluates the sensitivity, specificity, and repeatability of this method, and verifies its detection performance and applicability for PRRSV-C, PRRSV-HP, and PRRSV-NA in clinical samples. The aim is to facilitate the simultaneous detection of multiple virus strains, enhance detection efficiency, and reduce detection costs. This study provides a reference for the rapid identification and diagnosis of different PRRSV strains and offers a scientific basis and technical support for the effective monitoring and control of PRRSV.

## 2. Materials and Methods

### 2.1. Primers and Probes

At least ten genome sequences of the NSP2 genes from PRRSV-C, PRRSV-HP, and PRRSV-NA were procured from the NCBI database for subsequent analysis using SnapGene (Version 6.0.2). Through comparative analysis of the NSP2 genes across each strain, the most conserved regions were identified, facilitating the design of primers and probes via Beacon Designer software Version 7.9 (Premier, Charlotte, NC, USA). TaqMan probes specific to the PRRSV-C, PRRSV-HP, and PRRSV-NA strains were labeled with VIC, FAM, and ROX at the 5′ end, respectively, while the 3′ end quenchers were BHQ1 for PRRSV-C and PRRSV-HP strains, and BHQ2 for PRRSV-NA strains. The sequences of the primers and probes developed in this study are detailed in Table 1. The synthesis of primers and probes was carried out by Kunshan Proprobes Biotechnology Co., Ltd. (Kunshan, China).

### 2.2. Standard Plasmid

The target fragments of PRRSV-C, PRRSV-HP, and PRRSV-NA were individually amplified, synthesized, and subsequently cloned into the pUC57 vector. Quantification of the standard plasmids, namely pUC57-PRRSV-C, pUC57-PRRSV-HP, and pUC57-PRRSV-NA, was conducted by qubit fluorescence quantitative analyzer, with copy numbers calculated according to a specified formula [20]. For the construction of multiplex standard curves, each plasmid was diluted to a concentration of 3.0 × 10^7^ copies/μL and combined in equal volumes to achieve a final concentration of 1.0 × 10^7^ copies/μL for each plasmid. The resultant pooled plasmid solution was then subjected to a 10-fold serial dilution, spanning from 1.0 × 10^7^ to 1.0 × 10^0^ copies/μL, to generate the multiplex standard curves. The diluted plasmid samples were subsequently stored at −20 °C for future use.

### 2.3. Reaction Conditions of the Triplex TaqMan-qPCR

All qPCR reaction systems were set to a volume of 25 μL. AK Taq one-step RT-PCR Mix (Heat-labile UDG) was purchased from Fapon Biotechnology Co., Ltd. (Catalog No: MD103P, Dongguan, China), and 5×Neoscript Fast RT Premix Buffer was purchased from Zhuhai Biori Biotechnology Co., Ltd. (Catalog No: M5244, Zhuhai, China). After optimizing the reaction, the PCR reaction system is shown in Table S1. Amplification was performed on an ABI 7500 Real-time System (ABI, Waltham, MA, USA) using the following program: 55 °C for 5 min, 95 °C for 2 min 30 s, 45 cycles of 95 °C for 15 s, and 58 °C for 40 s. Fluorescence signal was automatically collected at the end of each cycle. All qPCR results were analyzed using 7500 System SDS Software Version 2.0.6 (Applied Biosystems, Foster City, CA, USA).

### 2.4. Construction of Standard Curves and Evaluation of Sensitivity

A 10-fold diluted pUC57-PRRSV positive plasmid, with concentrations ranging from 1.0 × 10^7^ to 1.0 × 10^0^ copies/μL, served as the template for triple fluorescence quantitative PCR amplification. This was conducted using the established TaqMan-qPCR method to generate amplification kinetics curves and assess the method’s sensitivity. Concurrently, the concentration of the standard plasmid was plotted on the horizontal axis against the cycle threshold (Ct value) on the vertical axis to construct the standard curve for the TaqMan-qPCR method, resulting in the derivation of the standard linear regression equation.

### 2.5. Evaluation of Specificity

To evaluate the specificity of the triplex TaqMan-qPCR method in the presence of other infectious viruses, the nucleic acid cDNA of eight common porcine viruses, namely PPV, TGEV, PRV, CFSV, ASFV, PEDV, RV, and PCV2, was included in the specificity assessment experiment. The pUC57-PRRSV-C, pUC57-PRRSV-HP and pUC57-PRRSV-NA mixed positive plasmid at a concentration of 1.0 × 10^3^ copies/μL served as the positive control for PRRSV, while ddH_2_O was used as the negative control. Following qPCR detection, the specificity of the detection method was thoroughly evaluated.

### 2.6. Evaluation of Reproducibility

Templates were prepared using the positive plasmids of pUC57-PRRSV-C, pUC57-PRRSV-HP, and pUC57-PRRSV-NA mixed in equal proportions to concentrations of 1.0 × 10^5^, 1.0 × 10^3^, and 1.0 × 10^1^ copies/μL. Fluorescence qPCR amplification was conducted employing the optimized reaction system and conditions, with three batches of repeated tests and four replicates for each dilution within each batch. The resulting Ct values were subjected to statistical analysis to determine the coefficient of variation both within and between groups, thereby assessing the reproducibility and stability of the method.

### 2.7. Comparative Analysis of the Established TaqMan-qPCR Assay Versus Commercial Kits

A total of 94 clinical samples were collected from PRRSV-positive farms, comprising blood, tissue, and throat swabs. These samples were analyzed using the established TaqMan-qPCR method and singlex qPCR method commercial kits from Ring Biotechnology Co., LTD (Catalog No: ZP016/ZP020/ZP066, Beijing, China). The detection performance of the developed triplex TaqMan-qPCR method was evaluated in comparison to commercial kits by calculating the relative sensitivity, defined as (true positive number/(true positive number + false negative number)) × 100%, the relative specificity, defined as (true negative number/(true negative number + false positive number)) × 100%, and the compliance rate, defined as ((true positive number + true negative number)/the total number) [21].

### 2.8. Clinical Sample Testing

A total of 1049 clinical samples were collected from pig farms in Hebei Province between 2023 and 2025. These samples were collected from pigs at different production stages, including suckling piglets, nursery pigs, finishing pigs, boars and sows. The main sample types consisted of whole blood, serum, tissue, throat swabs, environmental samples, semen, and anal swabs. Specifically, 300 samples were collected in 2023, 432 in 2024, and 317 in 2025. All samples were collected on-site by farmers, transported to our laboratory under cold-chain conditions, and stored at −80 °C until testing. Relevant information for all samples, including sampling time, sampling location, and sample type, was thoroughly documented.

RNA extraction was conducted on 300 μL of each sample or its elution using the CM-3200 fully automatic nucleic acid extractor (Yuanzhen Biopharmaceutical Co., Ltd., Baoding, China), followed by reverse transcription. The qPCR analysis was then performed on 5 μL of the extracted DNA using a triplex TaqMan-qPCR method developed in-house. Three types of pUC57-PRRSV standard plasmids served as positive controls, while ddH_2_O was employed as a negative control. A Ct value of less than 38 was deemed indicative of a positive result. Concurrently, we examined the prevalence of three lineages of PRRSV over the period from 2023 to 2025.

## 3. Results

### 3.1. Standard Curves and Evaluation of Sensitivity

**Figure 1:**
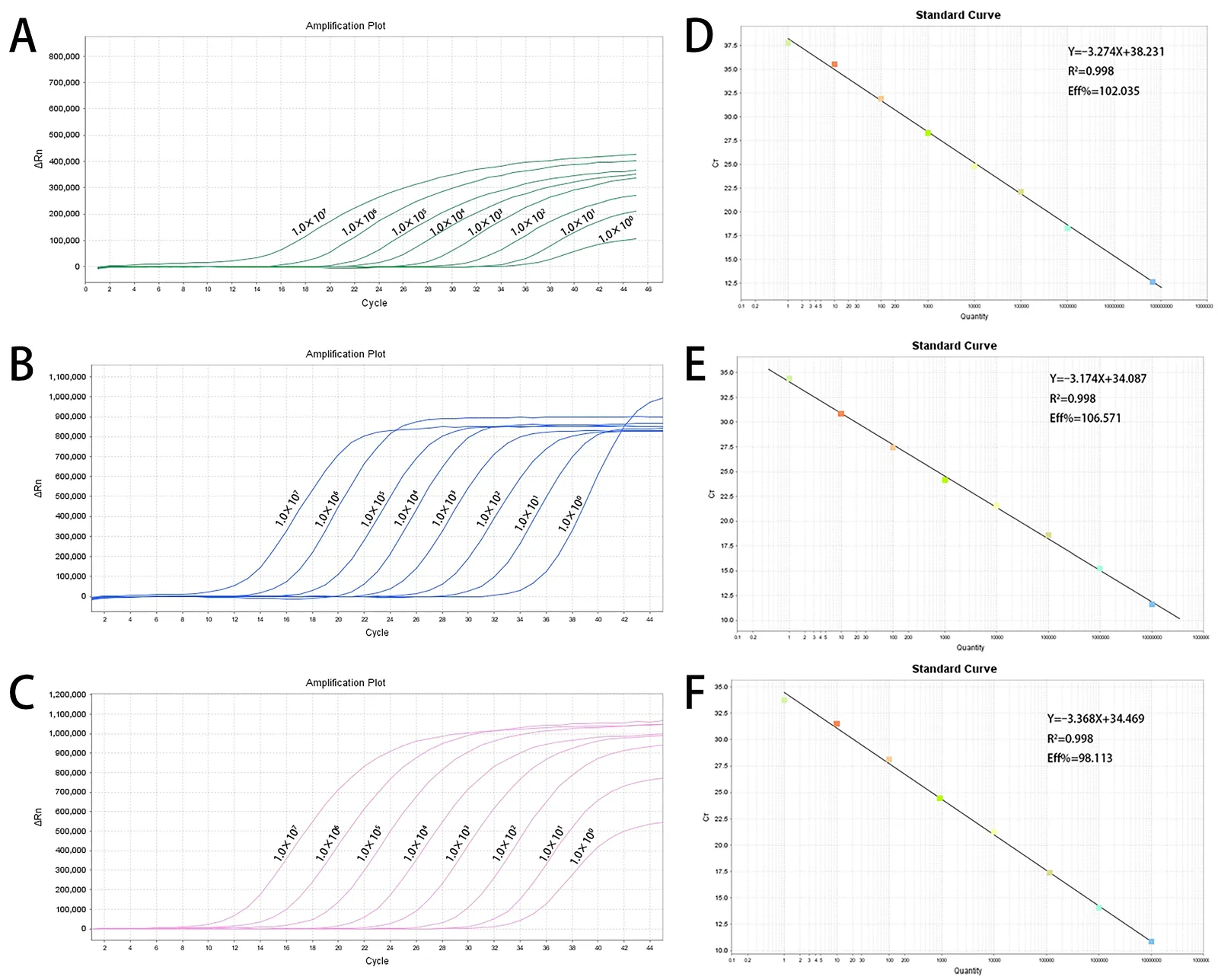
Sensitivity amplification curves and standard curves of the triplex fluorescent quantitative PCR. (**A**–**C**) The sensitivity amplification curves of nsp2 gene of PRRSV-C with FAM channel (**A**), PRRSV-HP with VIC channel (**B**), and PRRSV-NA with ROX channel (**C**). (**D**–**F**) Standard curves of NSP2 genes of PRRSV-C (**D**), PRRSV-HP (**E**) and PRRSV-NA (**F**).

The triplex qPCR assay exhibited excellent linear correlation and high amplification efficiency across a wide range of template concentrations. The LOD for all three targets was 1.0 × 10^0^ copies/µL using serially diluted standard plasmids (Figure 1A–C). It should be noted that the practical LOD in clinical matrices (e.g., serum, tissue homogenates) may be slightly higher due to variations in nucleic acid extraction efficiency and reverse transcription performance. Standard curves were automatically generated by the TaqMan qPCR instrument. The standard curve for the NSP2 gene of PRRSV-C was characterized by the equation Y = −3.274X + 38.231, with an R^2^ value of 0.998 and an efficiency of 102.035% (Figure 1D). Similarly, the standard curve for the NSP2 gene of PRRSV-HP was Y = −3.174X + 34.087, R^2^ = 0.998, and an efficiency of 106.571% (Figure 1E). For the NSP2 gene of PRRSV-NADC30-like, the standard curve was Y = −3.368X + 34.469, R^2^ = 0.998, with an efficiency of 98.113% (Figure 1F). All amplification efficiencies fell within the acceptable range of 95–110% for qPCR assays, and the high R^2^ values confirmed strong linearity between template concentration and Ct values, indicating reliable quantitative performance across the tested dilution range.

### 3.2. Specificity

**Figure 2:**
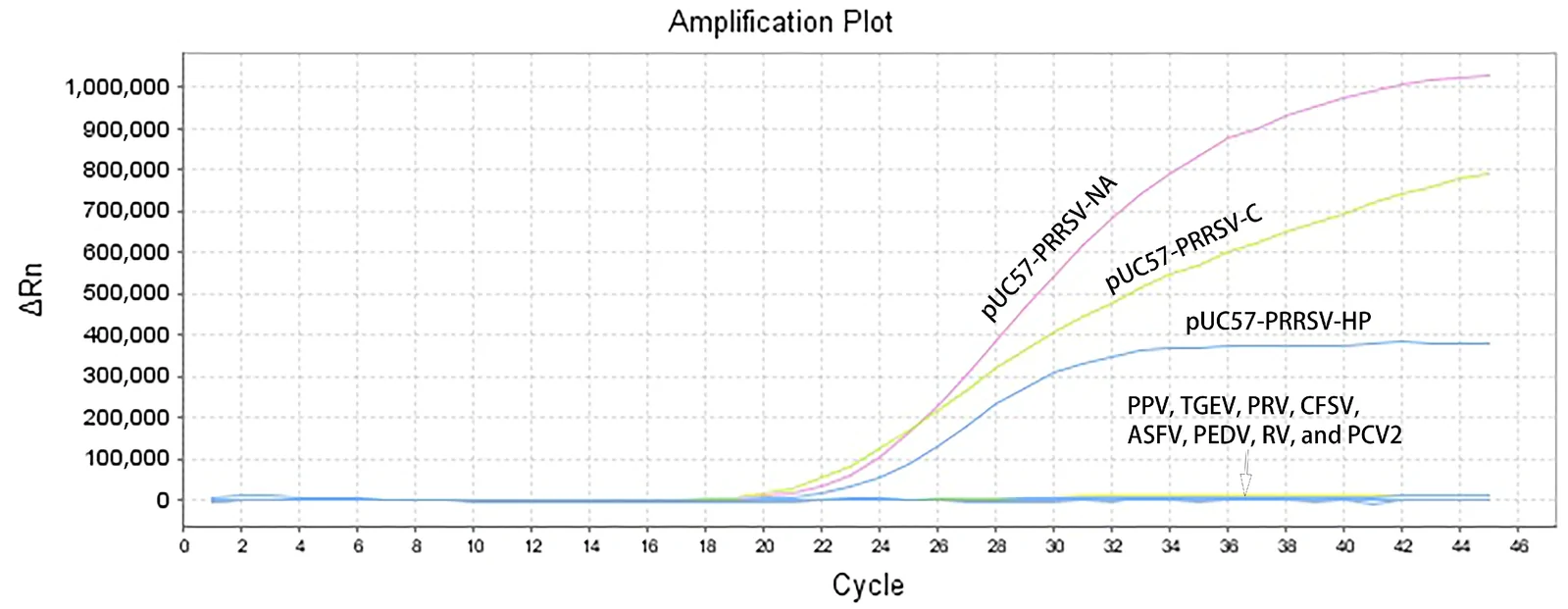
Amplification curves of specificity testing among porcine viruses.

The triplex qPCR assay showed high specificity for the three PRRSV lineages. Distinct amplification signals were detected only in reactions containing the corresponding positive plasmid mixtures of PRRSV-C, PRRSV-HP, and PRRSV-NA, while no amplification curves were observed for any other porcine infectious pathogens included in the specificity panel (Figure 2). The absence of cross-reactivity among the three targets and with non-target pathogens ensures accurate differential detection in clinical samples with potential co-infection and minimizes the risk of false-positive results.

### 3.3. Reproducibility

The reproducibility of the triplex assay was evaluated by calculating intra-assay and inter-assay CV values. As shown in Table 2, intra-assay CV values ranged from 0.46% to 1.93%, and inter-assay CV values ranged from 0.45% to 3.25%. All CV values were below 5%, which meets the performance criteria for quantitative molecular diagnostic methods, indicating that the established assay has excellent repeatability for routine laboratory use.

### 3.4. Comparison with Commercial Single-Plex qPCR Kits

As detailed in Table 3, the analysis of clinical samples using the developed triple TaqMan-qPCR and commercial kits demonstrates that the relative sensitivity of the three targets is 100%, suggesting equivalence in sensitivity to commercial kits. Furthermore, the relative specificity and compliance rates of the triplex qPCR method, as established in this study, exceed 98% compared to commercial kits, underscoring its potential as a viable alternative to existing commercial qPCR methods.

### 3.5. Clinical Sample Testing

**Figure 3:**
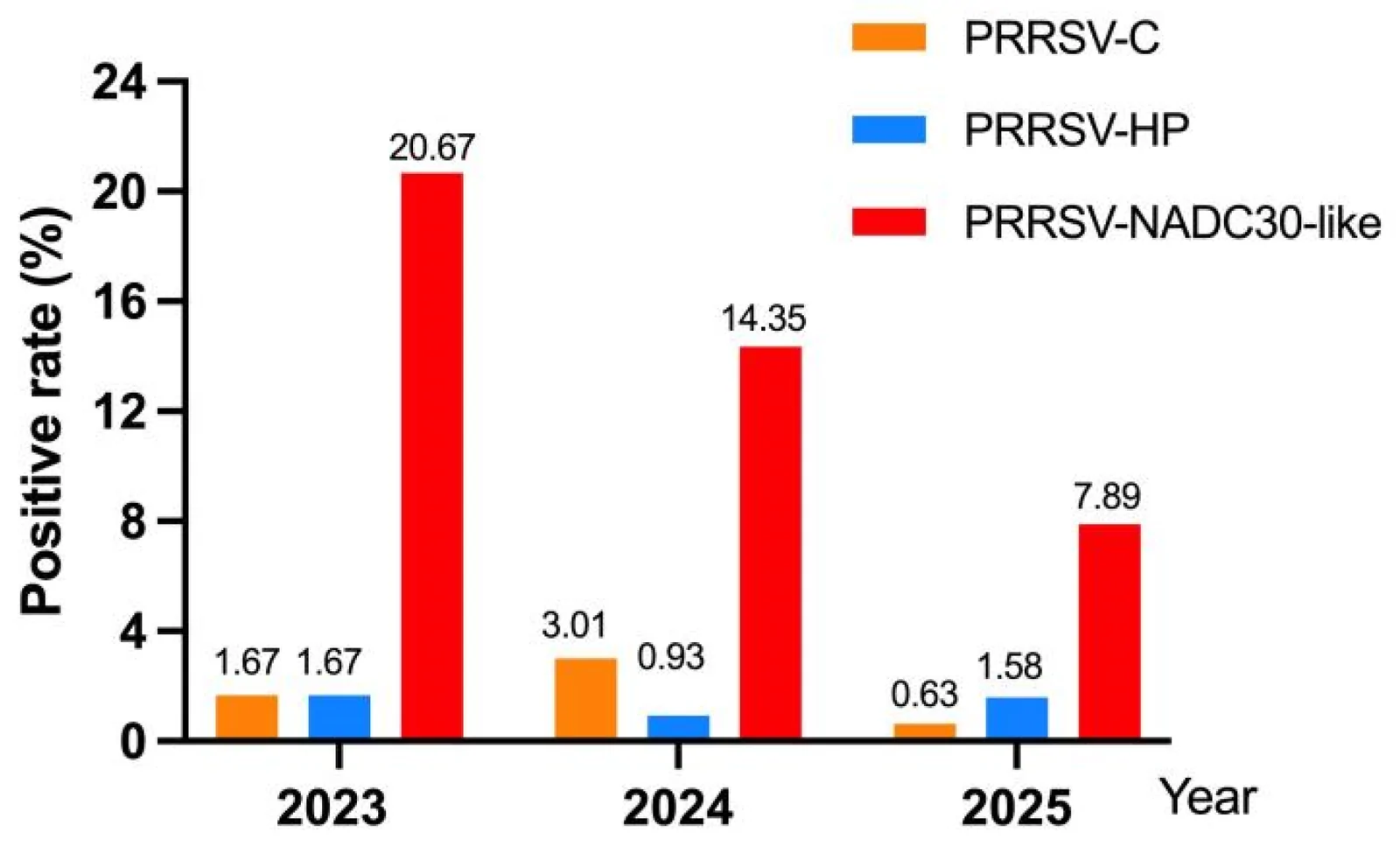
Positive rates of PRRSV-C, PRRSV-HP, PRRSV-NA in 2023–2025 in Hebei province.

To evaluate the field applicability of the established triplex qPCR assay, we tested clinical samples from Hebei Province (2023–2025) to preliminarily characterize the local epidemiological profile of different PRRSV-2 lineages. As shown in Figure 3, the positive detection rate of the NADC30-like lineage exhibited a continuous year-on-year decline across the study period: from 20.67% in 2023 to 14.35% in 2024, and further to 7.89% in 2025. Despite this downward trend, the data indicate that NADC30-like strains are currently the predominant circulating PRRSV-2 strains in Hebei Province. This decline may be attributed to the gradual improvement of biosecurity systems on local pig farms, the application of lineage-matched vaccines, and the natural attenuation of viral epidemic intensity after years of circulation. In contrast, PRRSV-C and PRRSV-HP strains maintained consistently low prevalence levels over the three years. The positive rates of PRRSV-C were 1.67%, 3.01% and 0.63% in 2023, 2024 and 2025, respectively, while the rates for PRRSV-HP were 1.67%, 0.93% and 1.58%, respectively. The consistently low prevalence of these two lineages corresponds with the broader epidemiological landscape of PRRSV in China. This situation is predominantly attributed to the prolonged and widespread use of existing commercial vaccines across the nation, which have successfully curtailed the circulation of both classical and highly pathogenic strains. Collectively, these clinical detection results validate the triplex qPCR assay as a dependable method for testing field samples and differentiating lineages.

## 4. Discussion

Due to the significant genetic diversity of PRRSV, it is primarily categorized into PRRSV-1 and PRRSV-2, with PRRSV-2 encompassing various lineages such as PRRSV-C, PRRSV-HP, and PRRSV-NA. The development of qPCR methods capable of concurrently detecting and differentiating these distinct genotypes and lineages holds substantial clinical and epidemiological importance. Researchers have successfully developed various multiplex qPCR detection methods by designing specific primers and probes targeting conserved regions of diverse viral genomes, and some have been commercialized. For instance, one study introduced an RT-qPCR method utilizing SYBR Green I dye, capable of simultaneously identifying PRRSV-1, PRRSV-2, PRRSV-HP, and PRRSV-NA. This method demonstrated LODs of 10^2^ copies/μL for PRRSV-1 and 10 copies/μL for PRRSV-2, PRRSV-HP, and PRRSV-NA, exhibiting high specificity and repeatability [22]. A more advanced approach involves the development of an RT-qPCR based on TaqMan probes, which employs four sets of primers and probes targeting the conserved regions of the PRRSV ORF7 and NSP2 genes. This method facilitates the simultaneous detection and typing of PRRSV-1, PRRSV-2-C, PRRSV-2-HP, and PRRSV-2-NA, with LODs as low as 12 copies/μL [23]. Additionally, a quadruplex RT-qPCR method developed by Ye et al., targeting the predominant PRRSV-2 lineages circulating in China (NADC30-like, HP-like, and NADC34-like), has demonstrated exceptional performance, achieving an LOD of 3 copies/μL [24]. This study established a more sensitive multiplex qPCR method based on optimizing primer sequences and reaction systems, achieving a detection sensitivity as low as 1 copy/μL.

Current detection targets for PRRSV include ORF5, NSP2, ORF7, and M genes. The GP5 protein, encoded by the ORF5 gene, serves as the principal envelope glycoprotein of PRRSV and represents a critical locus for viral genetic diversity and immune selection pressure. Consequently, it has been extensively utilized as a primary target for the molecular detection and typing of PRRSV over an extended period [22]. The RT-qPCR method, which targets the ORF5 gene, effectively differentiates between PRRSV-1 and PRRSV-2, and further identifies circulating lineages within PRRSV-2, such as PRRSV-C, PRRSV-HP, and PRRSV-NA [23]. Nevertheless, the high variability of the ORF5 gene presents a significant limitation for its application as a detection target. Research indicates that the antigenic epitope regions of the GP5 protein frequently undergo amino acid mutations and deletions, potentially rendering primers and probes designed based on specific ORF5 sequences ineffective in detecting newly emerging variant strains, thereby leading to false-negative results [25]. As a supplementary component of this study, targeted ORF5 sequencing was conducted on 31 positive samples. Nucleotide sequence identities of the ORF5 gene spanned 79.5–100.0%, and marked variation in sequence identity was evident across separate phylogenetic clades. The ORF7 gene and its adjacent M gene represent the most conserved regions within the PRRSV genome and are frequently utilized as targets for developing broad-spectrum PRRSV detection methods designed to concurrently identify both PRRSV-1 and PRRSV-2 genotypes [23,26]. In contrast, the NSP2 gene constitutes one of the most variable regions of the PRRSV genome, encoding a protein characterized by a highly variable region that frequently undergoes discontinuous amino acid deletions, insertions, or mutations. These variations serve as critical molecular markers for differentiating between distinct PRRSV lineages [27]. For instance, the HP-PRRSV strains prevalent in China are typically associated with characteristic discontinuous deletions of 30 amino acids within the NSP2 region, whereas NADC30-like strains exhibit a pattern of 131 amino acid discontinuous deletions [11,28]. Utilizing the differences in deletion patterns within the highly variable region of the NSP2 gene, researchers have developed a multiplex qPCR method capable of simultaneously detecting PRRSV-2 and differentiating between NADC30-like, HP-PRRSV-like, and NADC34-like strains [24]. The primary advantage of this method is its reliance on the relative stability of the NSP2 deletion pattern, which is closely linked to the classification of viral lineages.

Nevertheless, due to viral recombination and evolution, NSP2 deletion patterns have exhibited increasing complexity. Research indicates that recombination events among different lineages occur frequently, rendering NSP2 deletion patterns insufficient as exclusive molecular markers for each lineage [29]. Consequently, optimized multi-target combination strategies are often employed, integrating both conserved and specific targets to enable comprehensive detection of PRRSV, genetic typing, and preliminary virulence assessment [30]. The approach developed in this study serves as a complementary method to the existing qPCR technique targeting the M gene in our laboratory, aligning with the strategy proposed by Tao et al. [30]. The integration of the two methodologies facilitates an initial screening for the prevalence of PRRSV, while subsequently focusing on variations such as NSP2 for detailed typing. This approach constitutes a “conservative screening + variation typing” strategy, aimed at achieving both sensitive and specific detection objectives. Furthermore, in follow-up analyses within this study, two PRRSV isolates were obtained from the positive samples, both of which belong to the PRRSV-NA lineages. Whole-genome sequencing analysis indicated that the genomic backbone of these two isolates was predominantly derived from NADC30-like strains, with a minor proportion of genomic fragments inserted from CH-1a and JXA1 strains. Consequently, given the frequent inter-lineage recombination of PRRSV, whole-genome sequencing remains crucial for analyzing complex recombinant isolates, despite the developed detection assay’s ability to provide accurate and specific genotyping based on the NSP2 gene region. Complementary whole-genome sequencing analysis will enhance the comprehensive elucidation of the evolutionary mechanisms underlying PRRSV.

Since the introduction of NADC30-like strains into China around 2013, there have been substantial shifts in their epidemiological status, with these strains now supplanting PRRSV-HP as the predominant lineage in the country [6]. Epidemiological surveillance data indicate that the NADC30-like strain exhibits a broad epidemic distribution and has emerged as the dominant strain in several China provinces. For instance, surveillance conducted from 2021 to 2023 in Sichuan Province revealed a detection rate of 74.18% for NADC30-like strains, significantly surpassing other lineages [6]. In a similar vein, surveillance conducted in Fujian Province from 2023 to 2024 revealed that NADC30-like strains constituted 45.95% of PRRSV-2 positive samples [29]. These findings collectively suggest that the NADC30-like strain has rapidly disseminated and extensively colonized pig populations in China, thereby forming the predominant component of the current PRRSV epidemic landscape. The study’s results further demonstrate that the detection rate of NADC30-like strains significantly surpasses that of PRRSV-C and PRRSV-HP. Despite the predominance of NADC30-like strains in Chinese pig populations, PRRSV-C and PRRSV-HP have not been entirely eradicated, persisting in certain pig farms and regions, and contributing to the intricate epidemic scenario of PRRSV in China. Given that the clinical evaluation was limited to samples from Hebei Province with a modest sample size, the epidemiological findings reported here are preliminary and region-specific. Larger multi-regional surveillance efforts will be undertaken in future work to validate the broader applicability of this assay.

## 5. Conclusions

In summary, this study established a triplex qPCR method capable of simultaneously identifying PRRSV-C, PRRSV-HP, and PRRSV-NA, providing a powerful technical tool for rapidly and accurately identifying the circulating PRRSV lineages in pig populations and formulating targeted immunization and prevention strategies.

## Figures and Tables

**Table 1:** Sequences of primers and probes.

Primers and Probes	Sequence (5′ → 3′)
PRRSV-C-F	AACGAAGCCTGTCAAGAGCTTG
PRRSV-C-R	YCGGGAGATCAAAGGGGCTAC
PRRSV-C-P	VIC-ACAGCMAAATCTTCCCAACYGTTA-BHQ1
PRRSV-HP-F	GTTCCTGCACCGCGTAGA
PRRSV-HP-R	AGGATRCCCATGTTCTGC
PRRSV-HP-P	FAM-CTGTGACAACAACGCTGACGCACCA-BHQ1
PRRSV-NA-F	TGGAYACCTCYTTTGATTGGRA
PRRSV-NA-R	CACRACAGTGACTGGAGCRTGACACT
PRRSV-NA-P	ROX-CAGTTYCGCTGCTTGARCAGCCACT-BHQ2

**Table 2:** Intra-assay and inter-assay reproducibility test of the triplex TaqMan-qPCR.

Target	Template Concentration (Copies/μL)	Intra-Assay Variation			Inter-Assay Variation		
		Average Value	Standard Deviation	CV	Average Value	Standard Deviation	CV
PRRSV-C	10^5^ copies/μL	17.64	0.28	1.57%	17.22	0.45	2.63%
	10^3^ copies/μL	23.71	0.11	0.46%	23.29	0.35	1.50%
	10 copies/μL	31.37	0.34	1.10%	31.08	0.80	2.58%
PRRSV-HP	10^5^ copies/μL	18.04	0.11	0.61%	18.04	0.08	0.45%
	10^3^ copies/μL	24.39	0.14	0.59%	24.20	0.20	0.82%
	10 copies/μL	32.41	0.46	1.42%	32.26	0.41	1.26%
PRRSV-NA	10^5^ copies/μL	12.50	0.24	1.93%	12.61	0.41	3.25%
	10^3^ copies/μL	22.35	0.33	1.46%	22.27	0.33	1.48%
	10 copies/μL	29.79	0.33	1.11%	29.74	0.28	0.95%

**Table 3:** Comparison between the TaqMan-qPCR method and commercial qPCR kits for PRRSV-C, PRRSV-HP and PRRSV-NA detection.

Target	True Positive	False Positive	False Negative	True Negative	Total	Relative Sensitivity	Relative Specificity	Compliance Rate
PRRSV-C	21	1	0	72	94	100%	98.63%	98.94%
PRRSV-HP	14	0	0	80	94	100%	100%	100%
PRRSV-NA	17	1	0	76	94	100%	98.70%	98.94%

## Data Availability

The data are not publicly available due to privacy and/or ethical restrictions, but can be obtained from the corresponding author upon reasonable request via e-mail.

## Supplementary Materials

The following supporting information can be downloaded at https://www.mdpi.com/article/10.3390/ani16172642/s1, Table S1: Reaction System of the Triplex qPCR.
