# Supplementary material for: Development and Application of a Triplex RT-qPCR Assay for Differentiating Major Lineages of Porcine Reproductive and Respiratory Syndrome Virus
Source: Animals (Basel). 2026 Aug 23;16(17):2642. doi: 10.3390/ani16172642 (PMC13564301; doi:10.3390/ani16172642)
Supplement: Supplementary file 1 [file animals-16-02642-s001.zip › animals-4459900-supplementary.pdf]

Table S1. Reaction System of the Triplex qPCR

Reaction components	Volume
PRRSV-C-F	0.7 μL
PRRSV-C-R	0.7 μL
PRRSV-C-P	0.6 μL
PRRSV-HP-F	0.5 μL
PRRSV-HP-R	0.5 μL
PRRSV-HP-P	0.3 μL
PRRSV-NA-F	0.5 μL
PRRSV-NA-R	0.5 μL
PRRSV-NA-P	0.4 μL
AK Taq DNA Polymerase	0.4 μL
MultiScript Reverse Transcriptase II	0.8 μL
RNasin (40 U/μL)	0.25 μL
Heat-labile Uracil-DNA	0.25 μL
5×AK RT buffer	5 μL
Solution I（10×）	2 μL
dUTP Mix（20 mM）	0.4 μL
Taq enzyme dilution buffer	0.9 μL
ddH_2O	5.3 μL
Template	5 μL
Total volume	25 μL
